# Increasing the demand for childhood vaccination in developing countries: a systematic review

**DOI:** 10.1186/1472-698X-9-S1-S5

**Published:** 2009-10-14

**Authors:** Beverley Shea, Neil Andersson, David Henry

**Affiliations:** 1 CIETcanada, 1 Stewart Street #319, Ottawa, Canada; 2 Centro de Investigación de Enfermedades Tropicales (CIET), Universidad Autónoma de Guerrero, Calle Pino s/n, Acapulco, México; 3 Institute for Clinical Evaluative Sciences, Toronto, Canada

## Abstract

**Background:**

Attempts to maintain or increase vaccination coverage almost all focus on supply side interventions: improving availability and delivery of vaccines. The effectiveness and cost-effectiveness of efforts to increase demand is uncertain.

**Methods:**

We performed a systematic review of studies that provided quantitative estimates of the impact of demand side interventions on uptake of routine childhood vaccination. We retrieved studies published up to Sept 2008.

**Results:**

The initial search retrieved 468 potentially eligible studies, including four systematic reviews and eight original studies of the impact of interventions to increase demand for vaccination. We identified only two randomised controlled trials. Interventions with an impact on vaccination uptake included knowledge translation (KT) (mass media, village resource rooms and community discussions) and non-KT initiatives (incentives, economic empowerment, household visits by extension workers). Most claimed to increase vaccine coverage by 20 to 30%. Estimates of the cost per vaccinated child varied considerably with several in the range of $10-20 per vaccinated child.

**Conclusion:**

Most studies reviewed here represented a low level of evidence. Mass media campaigns may be effective, but the impact depends on access to media and may be costly if run at a local level. The persistence of positive effects has not been investigated. The economics of demand side interventions have not been adequately assessed, but available data suggest that some may be very cost-effective.

## Background

Routine childhood vaccination is an important prevention strategy with largely proven impact. The WHO claimed that, in 2001, childhood vaccination prevented 61% of deaths from measles, 69% of tetanus deaths, 78% of pertussis deaths, 94% of diphtheria deaths and 98% of polio deaths [1].

Despite this impressive potential, childhood vaccination coverage is stagnating or even deteriorating in some areas in South Asia and large parts of Africa [2]. Responses to the deteriorating coverage have focussed almost entirely on supply side improvements, including the development of new vaccines and extension of existing delivery services [3,4]. Much less is known about what increases *demand *for vaccination and the uptake from the users' perspective. A recent systematic review of 60 studies of evidence on improving routine vaccination programs in developing countries found only three studies that increased demand for vaccination [5].

In preparation for a cluster randomised trial of knowledge translation (KT) in the Balochistan province in Pakistan [6], we reviewed the literature on efforts to stimulate demand for routine childhood vaccination. The trial aimed to increase the demand for vaccination without relying on improvements or extension of the health services offered by the government.

## Methods

We developed an *a priori *protocol. Initial literature scans identified published systematic reviews of the childhood vaccination literature. Two reviewers (BS, DH) scrutinized these to identify relevant studies, and these were included in the review. We then searched for primary studies published since 2004 (the most recent literature update from existing reviews) using MEDLINE, POPLINE, ECONLIT, EMBASE, CINAHL and the Cochrane Library. We did citation searches on relevant articles using SCOPUS up to the end of September 2008. Search terms varied by database (details are available from the authors) and included vaccin*, immuni*, econom*, cost*, benefit* 'developing countries' and the names of countries that are categorised as 'low income' by the World Bank. We did not limit the searches by study type. These searches yielded 71,796 citations between 2004 and 2008.

**Figure 1:**
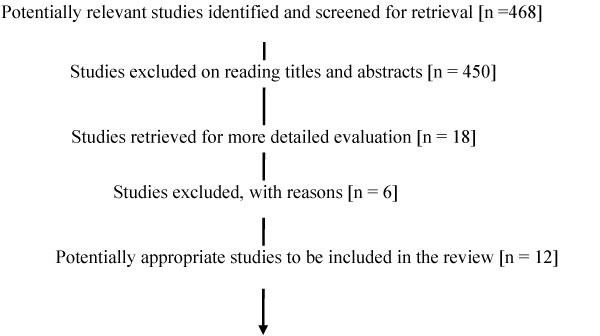
**Flow Chart for included and excluded systemic reviews and primary studies**.

Combinations of search terms identified a smaller number of potentially relevant titles from each database: MEDLINE (335), EMBASE (106), and CINAHL (27). There was considerable overlap between the retrieval lists for these databases. Two readers (BS, DH) examined all titles and identified 12 articles describing community-based interventions that might increase the demand for childhood vaccination (see Figure 1).

We excluded studies of exclusively supply side initiatives and those from developed countries. We included evaluation of national vaccination campaigns and the impact of community health workers when reports included a description of activities that seemed designed to increase demand for childhood vaccination. We evaluated studies on media campaigns, focus groups and microfinance programs and incentives. We also included studies containing cost data, and those from developing countries. Two authors (BS, DH) read the full text copies of reports of these studies.

Where interventions described a clear communication strategy we attempted to categorise it as 'knowledge transfer' - a unidirectional process where research is conceptualized and conducted, and the results are then made available to the end-users - or 'knowledge translation', a process that involves active and conscious participation of knowledge translators and knowledge recipients - in this case the parents of children. We confined our literature retrieval to studies that provided quantitative estimates of the impact of 'demand side' interventions. If they used mixed methods, including qualitative techniques, they were included; but we did not extract or evaluate qualitative data. We sought studies with the highest levels of methodological rigour, following an evidence based approach [7]. In the case of published systematic reviews and meta-analyses we assessed methodological quality using the AMSTAR, a validated instrument [8,9]. We conducted formal assessment of randomised trials using the SIGN 50 instrument [10]. However, the broad range of the other study types required an informal approach to quality assessment and precluded any attempt at data pooling (meta-analysis).

## Results

### Review of reviews

Our search identified seven potentially relevant systematic reviews [3,5,11-15] and colleagues reviewed evidence on a broad range of perinatal and neonatal interventions, including tetanus toxoid to prevent neonatal or maternal tetanus. They did not consider childhood vaccinations. Edejer and colleagues reviewed the cost-effectiveness of several interventions in children under the age of five [12]. The interventions included measles vaccination, but not interventions to increase the uptake of vaccines. The Cochrane Review of the impact of lay health workers [12] included three randomised trials of interventions to increase vaccine coverage. These were conducted in developed countries and one was concerned with adult vaccination practices. All were excluded from this review.

Table 1 summarises the other four systematic reviews, all of which are relevant to childhood vaccination. Pegurri and colleagues [3] reviewed published studies up to 2001. They examined supply side interventions (bringing services closer to the community), demand side interventions (door to door canvassing) and interventions that had features of both supply and demand (mass vaccination campaigns). The increase in full vaccine coverage rose from the baseline 34% by an average of 27%.

**Table 1:** Characteristics of systematic reviews included in this study.

Review	Literature search and eligibility criteria	Characteristics of studies	Population	Outcome
Pegurri et al 2004 [3]	To Dec 2001/published and grey literature Adequate description of the intervention and either time series or 2 population groups	Evaluated Cost or Cost Effectiveness	Children <5 y in developing countries	% increase in coverage
Batt 2004 [13]	Search up to May 2003 Extensive grey literature including interviews with 28 international experts searching large databases, and a comprehensive search and retrieval of information from a large number of organizations e.g.WHO, GAVI, UNICEF	Evaluated Cost, Effectiveness and Cost Effectiveness	Children <5 y in developing countries	% increase in vaccine coverage, cost and dollar cost per fully vaccinated child
Haines 2007 [14]	Existing published RCTs, Cochrane library, Grey literature sources, references	Evaluated impact and cost effectiveness of Community health workers undertaking a range of tasks relevant to child survival goals	Children <5 in developing countries	% increase in coverage
Ryman 2008 [5]	Search up to Dec 2004 Extensive search in public and grey literature and they contacted 31 experts in the field	Effectiveness only	Routine vaccination in low and middle income countries	Change and FVC in children

*Items from the AMSTAR instrument are described in Additional file 1: A measurement tool to assess systematic reviews (AMSTAR).

Most studies showed improvement and the authors found no difference in effectiveness between 'supply side', 'demand side' and 'mixed' strategies. The economic analyses found estimates of costs per fully vaccinated child ranging from $0.9 with peer training in Indonesia to $245 for children under one year of age in an outreach program in Mauritania. Most estimates fell between $10 and $20 per fully vaccinated child. Channelling (door to door canvassing) and community health workers were both cost effective and both involved some degree of community interaction. The studies provided little information on duration of the improvement of community uptake of vaccinations. The review also highlighted the generally poor methods employed in the evaluation of these key public health interventions.

The review by Batt and colleagues [14] (Table 1) came from the same institution as that of Pegurri et al and used similar methods [3]. The added value was an extraordinarily detailed search and retrieval of the grey literature. This included searches of institutional document centres (WHO and UNICEF) and interviews with experts from key institutions around the world. The review identified 24 relevant intervention studies, 15 at national level. The 'demand-side' interventions involved education of communities and improving awareness of 'missed opportunities'. Studies of mass media, health worker education and community education claimed large effect sizes - absolute increases in proportions of full vaccine coverage (FVC) of up to 50%. The average effect of all interventions was to increase FVC from 44% to 64%, an increment similar to that estimated by Pergurri [3]. Most of the estimates of cost-effectiveness ratios fell below $50 per additional fully vaccinated child. There was no clear indication of whether any particular approaches to extending vaccine coverage were self-sustaining and led to long-term improvements.

Haines and colleagues [15] highlight the role of community health workers (CHW) in low income countries in undertaking interventions designed to improve child survival, including vaccination. CHW can be given practical training in delivery of key services and are cheaper to train and employ than doctors and nurses. The authors discuss arguments around CWHs as extensions of the health care system and their advocacy roles as agents for changing community behaviours. Some of this activity can be viewed as demand-inducing, although separating this from the supply side is difficult. The review considered a range of preventive and curative interventions that can involve CHWs, presenting results as a narrative. They point to the advantages of integrating vaccination programs with other interventions (distribution of impregnated bed nets for malaria and Vitamin A supplementation).

The recent review by Ryman [5] concentrated on efforts to strengthen routine vaccination services and used a broader search approach than did Pegurri [3] and Batt [14]. They retrieved papers published up to the end of 2004, including an extensive review of the grey literature. They excluded most of these candidate studies, however, because of methodological inadequacies. Their approach to study eligibility required a score over 60% on ratings of methodological quality. Quality criteria were explicit, but the threshold of 60%, measured by an un-validated instrument, adds an unspecified dimension. Only 25 papers met their criteria for inclusion and three studied KT to increase demand for vaccination. These are included in our review of individual studies (Table 2). Ryman [5] and colleagues comment that "mass communication campaigns have the potential to reach large numbers of people if access to the type of media... is good." Participation in an NGO credit (micro-finance) program increased vaccination coverage without the provision of additional services [5]. Training community members to provide information regarding vaccination and providing resource rooms did not increase overall vaccination coverage but improved the timeliness of vaccination.

**Table 2:** Characteristics of included studies.

Study ID	Participants	Study interventions	1) Evaluation Methods 2) Study quality	Study outcomes	Results
Loevinsohn 1987 [19]	Santa Rosa del Penon in the pacific northwest of Nicaragua	Mass vaccination campaigns; stationary clinics and mobile clinics with or without food supplementation as an incentive.	1) Measurement of attendance rates at mobile and stationary well child clinics. 2) Repeated surveys of clinic attendance; little detail of data collection or quality control; study used attendance rather than vaccination status as the outcome; lack of contemporaneous control group makes order effects possible.	Attendance at clinics; personnel time.	Regular mobile N = 425, 63.3% Mass vaccination campaigns N= 889, 77,1% Stationary with food N = 764, 94.1% Mobile with food N = 547, 99.2% Stationary clinics took up half as much healthy worker time as mobile clinics Person hours per village served MVC 8.5; Mobile clinics 38;Stationary clinics 19. Attendance declined in a linear fashion with distance from stationary clinics.
Cutts 1990 [20]	Mothers from Mozambique Maputo 210 children aged 12-23 months	Comprehensive and integrated intervention: Outreach teams visited in 3 consecutive monthly 'pulses'; communications system to inform villages about arrival of mobile teams. Training of representatives from grass roots organisations (ten-family leaders); development of community-based volunteers from grass roots organizations; Door-to-door canvassing. Health departments and Executive Councils formed intersect oral communities to organize activities with emphasis on mobilization	1) Cumulative BCG vaccination rates in the pulse project districts in 1984 (pre) and 1987 (post). 2) Difficult to apply rigorous study methods in a war zone; Post survey used EPI cluster sampling method; vaccination rates obtained from health cards; lack of contemporaneous control group makes order effects possible.	Comparison of coverage before and after program acceleration.	Measles vaccination 1985-1987 Beira (1% increase) Inhambane (13% increase) Tete (22% increase) Quelimane (31% increase).
Zimicki 1994 [16]	Philippines Pilot in Manila (1988) and nationally (1990) Mothers or permanent carers with children under 2 years	The mass-media element of the campaign was March-Sept 1990. It focussed on measles as a way of bringing mothers to health centres, mainly in urban areas. Four television and four radio advertisements were broadcast, and advertisements were printed in newspapers reminding people of vaccination day. Concentrated on the dangers of measles Other promotional materials included posters and bunting.	1) Two surveys of the carers of children aged < 2 years to measure a change in knowledge) and vaccination rates. A pre-post study of 60 health centres in the same areas. 2) National campaign, so parallel control group not possible; used a rigorous multi-stage cluster sampling with weighted analysis adjusted for clustering to standardise the 1990 sample to 1989 sample; lack of contemporaneous control group makes order effects possible.	12-23 month vaccine coverage (all 8 vaccines). 2-8 month vaccine coverage (at least 4 vaccines). Vaccination started on time and finished on time.	Mean number of vaccinations: Rate difference 53.6% (1989) to 64.5% (1990) (RD 10.9 (2.8-19.0) -starting on time (12.3 (1.5-23.1) -finished on time (24.0 (12.2-35.8) -appropriate early (8.5 (0-17.1) Exposure to the mass-media campaign and knowledge level RD2.32 (2.19-2.46) Increase knowledge increase vaccination (1989-1990) Absolute diff 0.77 (P < 0.0001).
Brugha 1996 [22]	Three towns in Eastern region of Ghana	Program of home visits during which parents or carers were advised to take the children to the next under five's clinic of their choice, and were given a referral note for the clinic. The intervention targeted parents of unvaccinated children. Up to 3 additional visits by a nurse over the next 6 months if the child did not complete vaccination.	1)Cluster randomised trial was conducted in the largest of the three towns. 2) Quality rating in Table 3.	Completed vaccination rates before and after the intervention using Road to Health cards, clinic records supplemented by maternal history.	Vaccination coverage rose from 59.5% to 86% in the intervention group compared with 60.7% to 66.7% in the control group. The difference in the increases in the intervention and control groups was statistically significant (P < 0.005). Vaccine coverage also rose in the other two towns that were also subject to the intervention but did not participate in the randomised trial.
Tulchinsky 1997 [21]	Communities in Hebron, the West Bank. 69 villages in Hebron and 20 in other areas	Village Health Rooms (VHR) implemented by village leaders. Each village health room is staffed by a female village guide selected by the village leaders and health office. The guide arranges mothers' visits. They also organize national vaccination days and have a teaching as well as a service role.	1) Data from individual patient records; household surveys to determine community basic demographic information and immunisation status. 2) Data collection based on secure records but lack of contemporaneous control group makes order effects possible.	Coverage utilization and improved health status; costs and program longevity	Coverage compared VHR with baseline data from the village household survey. 90% of children up to the age of 2 years had received measles or MMR and 96% had 3 or 4 doses of DPT. There was little changed from previous estimates but in the past children generally had vaccination delayed into the third year of life.
Amin 1997 [18]	Villages from 5 NGO regions in Bangladesh. 3,564 married women under the age of 50	NGO provision of small collateral-free area focused credit.	1) Cross sectional survey Multistage cluster sampling strategy; recruitment from villages where NGOs maintained rural credit programs and control areas where NGOs had no presence. 2) Parallel study of intervention and control areas; able to study credit recipients and non-recipients in program areas. Multivariate analyses to adjust for potential confounders.	Vaccination status amongst loanees and non-loanees from credit and non-credit areas	Mean age 29, av 3.1 children per household Last born vaccinated Total area 62.4% Credit program 67.8% non-member 58.8% Comparison area 49.4% Last born less than one Total area 66.7% Credit area 71.8% non-member 63.3% Comparison area 50.7% Infant mortality rates were lower in members of credit schemes in program areas.
Hutchinson 2006 [17]	People living in rural areas of Bangladesh	'Smiling Sun' communication program included a variety of important health-related messages. The delivery media included signboards, television drama series, television advertisements, radio spots, press ads in newspapers and local publicity. Messages related to maternal and child health, family planning and communicable disease control (incl vaccination).	1) Cross sectional survey using two-stage cluster sampling; correlation between exposure to the campaign and reported vaccination status was calculated. Extensive costing data collected. 2) National campaign, so parallel control group not possible. Used bivariate probit likelihood method to estimate program effectiveness.	Self-reported exposure to the 'Shining Sun' media campaigns and simultaneous self-reporting of key health-related behaviours (use of ante-natal care and use of childhood vaccinations). Cost-effectiveness analyses.	Mothers who recalled seeing Smiling Sun promotional material were more likely than those who did not to complete DPT vaccination (64% vs 48%). Marginal effectiveness remained positive after adjustment. Cost-effectiveness: National level data: $0.30/additional child vaccinated for measles and $0.36/additional child with DPT3. Local promotional activities: $32/additional child vaccinated for measles and $37/additional child vaccinated with DPT3.
Andersson 2009 [6]	Lasbela Pakistan parents of 12-23 months old	Three structured discussions with one in every ten thousand respondents. 1) Discussion showed findings about vaccine uptake from baseline survey. 2) Focused on the costs and benefits of childhood vaccination. 3) Focused on local action plans, including options for sharing transport and childhood costs.	1) Cluster randomised controlled trial. 2) Quality rating in Table 3.	Uptake of measles and full DPT vaccination.	Measles OR 2.20 (95% CI 1.2-3.88) DPT OR 3.36 (95% CI 2.03-5.56).

The quality of the four systematic reviews included in our review was mixed. All four performed comprehensive literature searches. Three reviews included grey literature. The authors provided reasons and justification for low scores, on several items of AMSTAR [8,9]. None of the included reviews had published *a priori *protocols nor had two reviewers check the selection and data extraction. No reviewer provided the list of included and excluded studies.

Only one review provided the characteristics of the included studies. Although the majority of reviewers assessed the scientific quality of the included primary studies, none of the reviewers included these measures of quality in their conclusions and recommendations. The majority of authors did not conduct a meta-analysis, largely because of the limitations of the primary studies in terms of consistent application of methodological and reporting standards. None of the reviewers addressed the potential competing interests of authors of the primary studies (Table 1).

In light of the broadly consistent findings of these published reviews, we extracted individual studies from them that reported the use of any information communication strategy to increase community demand for routine childhood vaccination. These reviews were extremely comprehensive in their coverage and covered the period up to the end of 2004. We augmented these with new studies published between January 1^st ^2004 and Dec 31^st^2007.

### Review of individual studies

From the literature search and scrutiny of the published systematic reviews we identified eight studies [6,16-22] that examined demand side initiatives to increase routine childhood vaccine coverage (Table 2). The studies can be categorised as those that involve KT and those that involve other approaches, including incentives.

### Knowledge translation interventions

Two studies considered the effect of mass media on uptake of vaccination. Zimicki and colleagues [16] described a national measles vaccination intervention in the Philippines that emphasized logistical information in the media (where and when vaccination was available), identifying a special day of the week as 'vaccination day'. The campaign consisted of four television and radio advertisements emphasizing the dangers of measles and the availability of free vaccination; newspaper inserts identified weekly vaccination days. Two surveys (before and five months after the media campaign) measured the impact of the campaign among mothers and carers of children under two years of age. The study included interviews with staff at 60 health centres and observations of 10 children who attended the health centres on vaccination days. The proportion of fully vaccinated children increased from 54% to 65% and the proportion of children aged 9-11 months who completed all vaccinations increased from 32 to 56%. There was improved knowledge about vaccination and some participants who recalled the campaign attributed some of their increased knowledge to this. Increased knowledge was associated with higher rates of completed vaccination. The interviews and observations at clinics revealed no changes in practices, so the authors attributed the increased uptake and completion of childhood vaccination to the mass media campaign. They argued that this was more likely in the targeted urban areas, with good access to radio television and newspapers. As with many countries where records are unreliable, this study used self-reporting, not vaccination records (discussed by the authors in Table 2). The evaluation of the Philippines program did not include an assessment of cost-effectiveness.

Hutchinson and others [17] described the 'Smiling Sun' multi-media campaign in Bangladesh between 2001 and 2003. A 26-episode television drama series featured recognisable local actors and intertwined drama and themes related to maternal and child health, family planning and communicable disease control (including vaccination). Episodes were followed by discussion and quizzes with prizes. The campaign included TV and radio promotions, posters, billboards, advertisements in newspapers and local publicity activities. At the community and clinic levels there were group meetings, rallies and loudspeaker announcements with campaign logos widely displayed. Two household surveys in 2001 and 2003 examined three outcomes: antenatal care, DPT3 and measles vaccination in children 12-35 months.

About one half of the respondents recalled seeing 'Smiling Sun' messages and, in the unadjusted analyses, this exposure was strongly associated with use of health services. Mothers recalling the campaign were more likely than those who did not to report DPT3 vaccination (64% versus 48%) and measles vaccination (79% versus 61%). The authors recognized that better off mothers were more in touch with the media and more likely to access health services. Their multivariate analysis attempted to test "pessimistic assumptions" under which they estimated the campaign increased vaccine coverage by 20% among those who could recall seeing promotional material. Although compatible with a positive impact, the evaluation design precludes firm conclusions about causality. They included detailed costing of the media campaign: the incremental cost effectiveness ratios were $0.30-0.36 per additional child vaccinated, compared with $32-$37 per additional child vaccinated for local publicity campaigns. The latter may be due to the high incremental costs of local promotional activities, including billboards and rallies.

Cutts and others [20] report on door-to-door canvassing for a vaccination program intended to accelerate the expanded program on vaccination in Mozambique. The initiative sought to increase uptake of vaccination services through three household visits by volunteers from grassroots organizations. Door-to-door canvassing established a 'census' of children and mothers requiring vaccination. They reported increased measles vaccination coverage between 1% and 31% in different towns that applied the scheme. They provide no cost estimates and do not try to quantify the cost transferred to the communities through the volunteers.

Tulchinsky and his colleagues [21] tested 'village-resource rooms' to provide a variety of preventive-oriented services (information about well baby visits and mass vaccination programs) in Hebrun, West Bank. The staff also carried out general health education activities in the community, aimed at improving the knowledge of new mothers. The intervention did *not *increase the vaccination uptake although they claimed the project met other goals: better access to and utilization of preventive health services. They do not provide costing data.

Andersson and colleagues [6] carried out a randomised controlled trial in Lasbela, one of the poorest districts of Pakistan. Interviewers contacted houses of children under the age of 60 months for the baseline and follow-up surveys. The intervention involved three rounds of discussion about vaccination with "opinion makers" in each of the randomly selected villages. These discussions included one in every ten potential respondents in the intervention clusters, not necessarily those who did respond to the questionnaires. The first round of discussion dealt with issues of access, the second discussed costs of illness and vaccination, and the third considered local options and solutions. The authors anticipated these discussions would roll on from the narrow contact base to other parents in the intervention clusters. Relying on self-reporting by mothers, they reported significantly higher measles and DPT vaccination uptake in intervention than in control clusters. The impact of this KT -- 20% increase in measles and 28.5% in full DPT -- indicated a high level of effectiveness of their three-visit evidence-based dialogue. The authors estimated the scheme could be expanded to cover the whole district for $9 per child included. Since the coverage with measles vaccination was low (around 50%) even with the intervention, this implies a cost of around $36-45 per additional fully vaccinated child.

### Incentives and other non-KT demand-side interventions

Collateral-free credit to poor women may improve their autonomy and capacity to care for their families. The interactions between thus empowered women could lead, in the logic of these initiatives, to social changes like increased uptake of vaccination. Amin and Li [18] looked at vaccination in villages where five NGOs provided microfinance. Their cross sectional survey found lower rates of infant mortality and modestly higher rates of vaccination among credit members than among non-member in the same area: last born vaccinated DPT3: 88.6% credit members, 82.1 non-members from the same district and 73.4% in non program districts. In the case of measles coverage: 67.8% for credit members, 58.8 for non-members in the same districts and 49.4% in non-program districts. Logistic regression analysis showed a significant relationship between credit membership and completion of vaccination. The study design did not permit firm conclusions about the causal nature of this intervention. There was also a strong relationship between vaccine coverage and infant death rates (< five years of age).

A second incentive study considered provision of food rather than credit. Loevinsohn and Loevinsohn [19] described a program in north-west Nicaragua where 19% of children under six years of age were malnourished. The mid 1980s saw establishment of well child clinics, some of them mobile. Stationary and mobile clinics introduced food supplements in 1985; every child who attended a clinic was entitled to 3.2 Kg of flour, 0.9 Kg of skim milk powder, one litre of cooking oil and three cans of chopped beef or pork. This was equivalent to three to five days of wages (or 3 to 7% of income if provided every three months). The authors found mobile clinics with food gifts achieved attendance rates of 99%, compared with 94% in stationary clinics with food, and 63.3% with regular mobile clinics. The assumption is that well child clinic attendance translates as completed vaccination. In the case of stationary clinics, attendance dropped with increasing distance to be travelled.

Brugha and Kevany [22] used a cluster-randomised design in Ghana to investigate the impact of an outreach intervention consisting of a home visit and referral note to a vaccination clinic followed by repeated visits by nurses to households of children who did not complete their vaccination schedule. Completed vaccination rose by 26.5% in the intervention group, compared with 6% in the control group (P < 0.005). Although this project appeared to involve little in the way of knowledge transfer or translation, the home visits by nurses aimed to increase uptake of vaccination, rather than to deliver vaccination in the home. We felt it important to include this study because it employed a rigorous evaluation methodology.

## Discussion

Our searches, including published systematic reviews and original studies, retrieved only eight published reports of controlled evaluations of the benefits and costs of interventions designed to increase demand for routine childhood vaccination. All of these studies showed some increases in uptake of vaccination, indicating that improvements of 10 to 20% are readily achievable.

The main approaches exemplified by these studies were knowledge transfer and provision of incentives and collateral-free credit. Having reviewed this body of data our main conclusion is that, despite their potential, and some encouraging results, interventions to increase demand for routine childhood vaccination have not been adequately investigated.

From a quantitative evidence based perspective, the level of evidence represented in the studies reviewed here is generally low, with a reliance on 'before and after' designs, employing repeated cross sectional surveys or retrieval of routinely collected data. It should be noted that the survey methods employed in some of these studies were of high quality and some authors made adjustments for variables that might act as confounders. However, the lack of concurrent control groups leaves most studies open to the possibility of 'order effects' (such as contemporaneous improvements in service provision) as an explanation for the findings. Attributing cause and effect is therefore difficult. This is disappointing, as the effect sizes seen in some studies were quite substantial. Furthermore, the variety of interventions and the lack of rigorous evaluations precluded any attempt to compare the effectiveness of the different approaches.

Thus, despite the crucial importance of the topic, interventions that increase demand for vaccination have received little research attention, in comparison with service enhancement approaches to increase vaccination coverage. Strategies that depend on use of the mass media do not lend themselves to evaluation by controlled trials as one cannot easily randomise exposure. They will not be effective if families are unable to access mass media - for instance in rural and remote areas. There is also the real issue of how long any effects of such campaigns last. To date, follow up times have been brief. We can speculate that the effects of mass media campaigns (on their own) will not last long compared with interventions that use education and reinforcement to engender basic changes in community attitudes to routine childhood vaccination.

Of course, the major challenge is to achieve such a sustained change. In our view this probably requires a shift from 'knowledge transfer' to 'knowledge translation', as defined in the Methods section. In this review we found no true example of an intervention that met the definition of 'knowledge translation'. But a realistic middle ground could be the type of village level discussions that we trialled in Pakistan [6]. This was facilitated by trained and paid fieldworkers with roll-on of the discussion after the team had left. In the Pakistan example, the main impact was anticipated from the social networking that followed the "paid intervention", in the form of three visits and discussions with opinion makers.

As illustrated in the studies reviewed here, demand side interventions can involve interventions that do not rely primarily on translation of knowledge. Collateral-free loans to women lead to empowerment and can result in collective actions, which can free up resources and overcome financial and logistical barriers to childhood vaccination. In this review the study by Loevinsohn [20] found in Nicaragua that food might provide an incentive to mothers to bring their children for vaccination. However, it is also likely that provision of food frees up other household resources and makes it easier for parents to organise vaccination visits. So it is equivalent to a financial payment.

The data on cost effectiveness were sparse, but indicated that demand side interventions are sometimes capable of providing incremental cost effectiveness ratios of less than $1 per additional fully vaccinated child. However, these low figures are very context specific as shown by the mass media campaign in Bangladesh [18,19] where the cost effectiveness ratios were between $30 and $40 per additional vaccinated child with local media campaigns, because of their higher net costs and lower coverage. There are many difficulties in attributing costs accurately to field activities. For instance, in volunteer based door-to-door interventions, like those in Mozambique, most of the true cost is absorbed by the volunteers, making the sustainability and reproducibility of the intervention questionable.

There are a number of important limitations in our work. One weakness of our review is that we did not update the extensive grey literature review undertaken by Batt and others (2004) [14]. This was a matter of resources - Batt and colleagues relied on an extensive series of interviews with key informants and we were unable to reproduce these. However, the grey literature reported in the systematic reviews included here was up-to-date and reflected complex interventions aimed at strengthening health systems, rather than the demand-side interventions reported in the published literature [14].

The work reported here was very much an update of previous reviews - and therefore depends on the foundational work done by others. Another limitation of our work was the impossibility of doing a meta-analysis, so we are left to present disaggregated results in the form of a narrative.

The overriding limitation of the review is the sparseness of the literature on increasing the demand for vaccination; this is of concern given the importance of the topic. To provide sustained effects demand side interventions will have to be integrated with other system-wide approaches and will not work unless procurement and supply are also addressed adequately.

## Conclusion

In the case of childhood vaccination, demand side interventions have been poorly investigated in developing countries. Even accepting the difficulties of carrying out research in this field, the available studies contribute only low levels of quantitative evidence. Recognising these limitations, the published studies reviewed here reported positive results, some claiming quite large increases in demand for childhood vaccination. However, the limited methodologies precluded any attempt to compare and contrast the effectiveness of different approaches. Mass media campaigns may be effective, but this will depend on access to media and may be costly if run at a local level. The studies reviewed here do not provide information on the duration of positive effects of mass media campaigns. The cost-effectiveness of demand side interventions has not been adequately assessed, but based on limited data some may prove to be very cost-effective.

## List of abbreviations used

CHW: Community health worker; FVC: Full vaccine coverage; KT: Knowledge Translation.

## Competing interests

The authors would like to declare two potential 'perceived risks of bias'. 1) Two of the authors (NA, BS) were involved in the Pakistan study, which is reviewed here. This review was conducted to assist with the development of the evidenced-based instruments used in the Pakistan trial. 2) Two of the authors (NA, BS) were involved in the development of AMSTAR and the third author (DH) assisted with the validation process.

Funding for this work came from the International Development Research Centre (IDRC) under grant 102172-007. The views expressed are those of the authors and do not necessarily reflect the views of IDRC.

## Authors' contributions

BS, DH and NA did the original conceptual development. BS and DH performed the literature search, data and quality assessments. BS, DH, and NA prepared and assisted with the manuscript.

**Table 3:** Methodological quality of systematic reviews and randomised trials primary studies included in this study.

Systematic review	AMSTAR	Quality Score (Percent of maximum)	Primary study	SIGN 50*	Percent quality
Pegurri et al 2004 [3]	1. No	18%	Brugha 1996 [22]	6/10	60%
	2. No				
	3. Yes				
	4. No				
	5. No				
	6. No				
	7. Yes				
	8. No				
	9. Can't answer				
	10. No				
	11. No				
Batt 2004 [14]	1. No	27%	Andersson 2008 [6]	6/10	60%
	2. No				
	3. Yes				
	4. Yes				
	5. No				
	6. No				
	7. Yes				
	8. Can't answer				
	9. Can't answer				
	10. No				
	11. No				
Haines 2007 [15]	1. No	18%			
	2. No				
	3. Yes				
	4. yes				
	5. No				
	6. No				
	7. No				
	8. No				
	9. No				
	10. No				
	11. No				
Ryman 2008 [5]	1. No	36%			
	2. No				
	3. Yes				
	4. Yes				
	5. No				
	6. Yes				
	7. Yes				
	8. No				
	9. Can't answer				
	10. No				
	11. No				

* Scores based on the first 10 items (internal validity) of the SIGN 50 Instrument http://www.sign.ac.uk/guidelines/fulltext/50/checklist2.html.

## Supplementary Material

Additional file 1 A measurement tool to assess systematic reviews (AMSTAR). Click here for file
